# Gastric-tube versus whole-stomach esophagectomy for esophageal cancer: A systematic review and meta-analysis

**DOI:** 10.1371/journal.pone.0173416

**Published:** 2017-03-07

**Authors:** Wenxiong Zhang, Dongliang Yu, Jinhua Peng, Jianjun Xu, Yiping Wei

**Affiliations:** Department of Cardiothoracic Surgery, The Second Affiliated Hospital of Nanchang University, Nanchang, China; University Hospital Oldenburg, GERMANY

## Abstract

**Objectives:**

To conduct a systematic review and meta-analysis of studies comparing the gastric-tube vs. whole-stomach for esophageal cancer in order to determine the optimal surgical technique of esophagectomy.

**Methods:**

A comprehensive literature search was performed using PubMed, EMBASE, ScienceDirect, Ovid MEDLINE, Cochrane Library, Web of Science, Google Scholar, and Scopus. Clinical trials that compared the gastric-tube versus whole-stomach for esophageal cancer were selected. The clinical endpoints included anastomotic leakage, anastomotic stenosis, reflux esophagitis, pneumonia, delayed gastric emptying, and thoracic stomach syndrome.

**Results:**

A total of 6 articles (1571 patients) were included. Compared to the whole-stomach approach, the gastric-tube approach was associated with a lower incidence of reflux esophagitis (95% confidence interval [CI]: 0.16 to 0.81, *p* = 0.01) and thoracic stomach syndrome (95% CI: 0.17 to 0.55, *p* < 0.0001). The rates of anastomotic leakage, anastomotic stenosis, pneumonia, and delayed gastric emptying did not significantly differ between the two groups.

**Conclusions:**

The gastric-tube esophagectomy is superior to the whole-stomach approach, as it is associated with a lower incidence of postoperative reflux esophagitis and thoracic stomach syndrome. Our findings must be validated in large-scale randomized controlled trials.

## Introduction

Esophageal cancer is a common type of cancer worldwide, and is associated with a high mortality rate [1, 2]. Surgical resection is the primary treatment for patients in the early and middle stages of esophageal cancer [3, 4]. The most suitable method of digestive tract reconstruction after esophagectomy for esophageal cancer is the anastomosis of the esophageal remnant with the stomach, as this ensures a reliable blood supply [5, 6]. Currently, both the gastric-tube and whole-stomach approaches are widely used for esophagogastric anastomosis [7, 8]. Some studies have concluded that the whole-stomach approach is superior to the gastric-tube approach, as it provides better protection of the submucosal vessels and can slightly increase gastric capacity [9, 10]. Furthermore, blood perfusion significantly decreases after tubular gastric surgery [11]. In contrast, other studies have shown that the anatomical structure of the gastric tube is more in line with physiological needs and could reduce the incidence of postoperative complications owing to the low anastomotic tension associated with this technique [12]. To determine the optimal technique, we conducted a systematic review and meta-analysis of studies investigating esophagectomy with gastric-tube and whole-stomach for the treatment of esophageal cancer.

## Materials and methods

### Search strategy

The study was conducted according to the Preferred Reporting Items for Systematic Reviews and Meta-Analyses criteria (PRISMA) as shown in S1 File. On August 10, 2016, we conducted an extensive literature search to identify all relevant studies published between January 1990 and August 2016, according to the Preferred Reporting Items for Systematic Reviews and Meta-Analyses. The following databases were scanned: PubMed, EMBASE, ScienceDirect, Ovid MEDLINE, Cochrane Library, Web of Science, Google Scholar, and Scopus. The search strategy was based on the combination of the following keywords or MeSH terms: (“esophagectomy” OR “oesophagectomy”) AND (“gastric tube” OR “tubular stomach”). In addition, we scanned the reference lists of the retrieved articles to identify relevant studies.

### Selection criteria

We used the following inclusion criteria: (1) clinical trials comparing the gastric-tube and whole-stomach for esophageal cancer, (2) studies including ≥10 patients in each group, and (3) the most recent study in the case of duplication of data in more than one article.

Reviews without original data, case reports, meta-analyses, letters, expert opinions, and animal studies were excluded.

### Data extraction

Data extraction was accomplished by two observers independently, using a standardized Excel form. Any disagreement was resolved the help of a third investigator. The recorded data included the following: first author, year of publication, study design, number of patients in each arm (gastric tube or whole stomach), and rate of postoperative complications (anastomotic leakage, anastomotic stenosis, reflux esophagitis, pneumonia, delayed gastric emptying, and thoracic stomach syndrome).

### Quality assessment of included studies

We assessed the methodological quality of the included studies by using the Newcastle-Ottawa Scale (NOS) in the case of non-randomized studies and the Jadad scale in the case of randomized controlled trials (RCTs). The 9-point NOS contains three main items: selection, comparability, and exposure. Studies that scored 8–9 points on the NOS were deemed to be of high quality, while those that scored 6–7 points were considered to be of medium quality [13]. The Jadad scale is a 5-point scale that evaluates the quality of studies on the basis of three items: randomization, masking, and accountability of all patients (withdrawals and dropouts). Studies that scored ≥3 points on the Jadad scale were considered to be of high quality [14].

### Statistical analysis

We used STATA 12.0 (StataCorp. LP, College Station, TX, USA) and Review Manager 5.3 (The Nordic Cochrane Centre, The Cochrane Collaboration, Copenhagen, Denmark) to conduct the meta-analysis. A *p*-value < 0.05 was considered to indicate statistical significance. Between-group differences in continuous variables were assessed using analysis of variance, while those in categorical variable were assessed using pooled relative risk with 95% confidence interval (CI). We used the *I*^*2*^ and Cochran Q statistics to evaluate heterogeneity among the studies. A random-effects model was adopted when significant heterogeneity was present (*p* ≤ 0.10 and *I*^*2*^ > 50%); otherwise, a fixed-effects model was used. The Egger test based on anastomotic leakage was used to assess potential publication bias.

## Results

### Literature search and quality assessments

**Fig 1:**
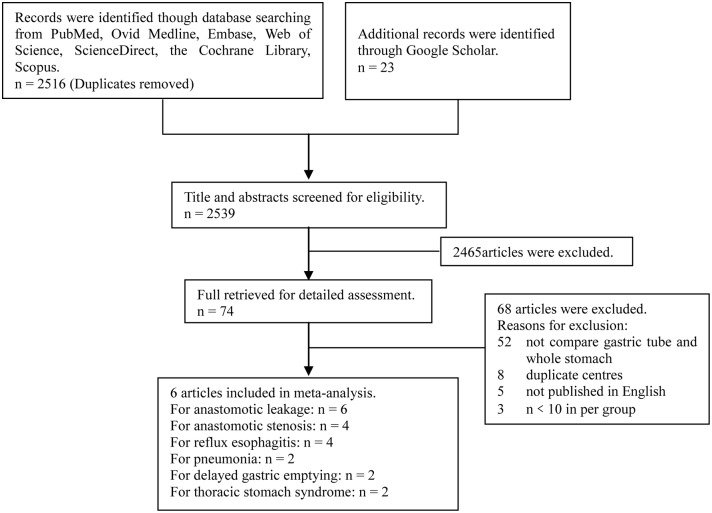
Flow diagram of study selection.

We initially identified 2539 publications from the database and reference-list searches. From these, we selected 6 studies with 1571 patients (826 in the gastric-tube group, 745 in the whole-stomach group) for the final analysis (Fig 1). Among the six studies, three were retrospective studies, and three were RCTs. Quality assessments using the NOS and Jadad scales showed that five studies were of good quality, and one study was of medium quality. The baseline characteristics of the included studies and the main evaluation indexes are shown in Table 1.

**Table 1:** Characteristics of the studies included in the meta-analysis.

Study		Groups	NO. of Patients (n)	Reflux esophagitis (n)	Thoracic stomach syndrome (n)	Anastomotic leakage (n)	Anastomotic stenosis(n)	Pneumonia (n)	Delayed gastric emptying (n)	Design	Quality (score)
1995	Collard [9]	Gastric tube	112	6		9	25			Retrospective	7
		Whole stomach	100	4		1	6				
2004	Tabira [15]	Gastric tube	22			5				RCT	3
		Whole stomach	22			1					
2009	Peng [16]	Gastric tube	120	6		2	12	3	0	RCT	4
		Whole stomach	120	31		4	15	15	15		
2013	Shu [12]	Gastric tube	453	23	15	25	42			Retrospective	8
		Whole stomach	397	44	39	37	39				
2015	Zhang [7]	Gastric tube	52	3	0	4	9	7	3	RCT	4
		Whole stomach	52	11	3	4	8	9	3		
2016	Zhang [17]	Gastric tube	67			1				Retrospective	8
		Whole stomach	54			3					

### Anastomotic leakage

**Fig 2:**
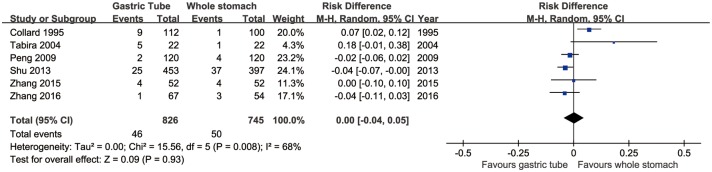
Forest plot of anastomotic leakage in the whole-stomach and gastric-tube groups.

All six articles evaluated the rate of anastomotic leakage. This rate did not significantly differ between the gastric-tube and whole-stomach groups (95% CI: -0.04 to 0.05, *p* = 0.93). However, significant heterogeneity was present across the studies (*p* = 0.008, *I*^2^ = 68%; Fig 2).

### Anastomotic stenosis

**Fig 3:**
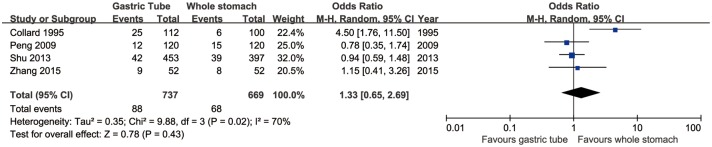
Forest plot of anastomotic stenosis in the whole-stomach and gastric-tube groups.

Four articles assessed the rate of anastomotic stenosis, which did not significantly differ between the two groups (95% CI: 0.68 to 2.69, *p* = 0.43). However, significant heterogeneity was present across the studies (*p* = 0.02, *I*^2^ = 70%; Fig 3).

### Reflux esophagitis

**Fig 4:**
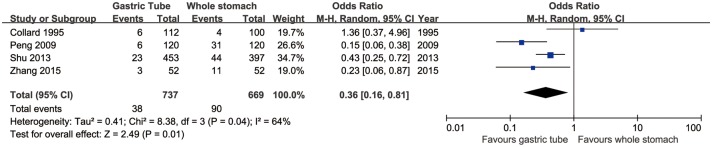
Forest plot of reflux esophagitis in the whole-stomach and gastric-tube groups.

Four articles reported the rates of reflux esophagitis. Reflux esophagitis was significantly more common in the whole-stomach group than in the gastric-tube group (95% CI: 0.16 to 0.81, *p* = 0.01). In addition, significant heterogeneity was present across the studies (*p* = 0.04, *I*^2^ = 64%; Fig 4).

### Pneumonia

**Fig 5:**
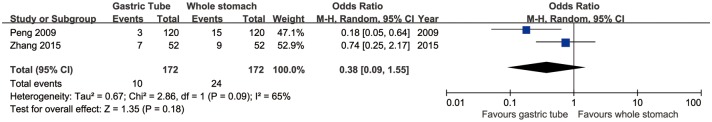
Forest plot of pneumonia in the whole-stomach and gastric-tube groups.

Two articles mentioned the incidence of pneumonia, which did not differ between the gastric-tube and whole-stomach groups (95% CI: 0.09 to 1.55, *p* = 0.18). However, significant heterogeneity was found across the studies (*p* = 0.09, *I*^2^ = 65%; Fig 5).

### Delayed gastric emptying

**Fig 6:**
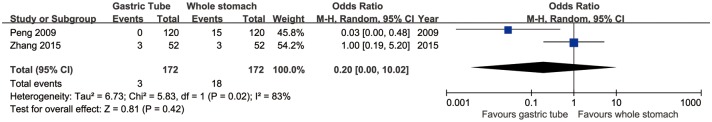
Forest plot of delayed gastric emptying in the whole-stomach and gastric-tube groups.

Two articles compared the rate of delayed gastric emptying between the gastric-tube and whole-stomach groups. This rate did not differ between the two study groups (95% CI: 0.00 to 10.02, *p* = 0.42). However, significant heterogeneity across the studies was detected (*p* = 0.02, *I*^2^ = 83%; Fig 6).

### Thoracic stomach syndrome

**Fig 7:**
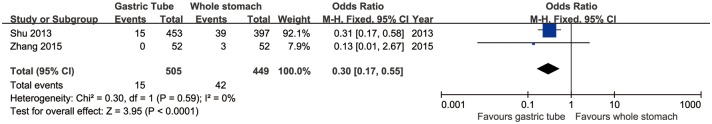
Forest plot of thoracic stomach syndrome in the whole-stomach and gastric-tube groups.

Two articles compared the rate of thoracic stomach syndrome between the two study groups. There was no evidence of heterogeneity between these two studies (*p* = 0.59, *I*^2^ = 0%). The incidence of thoracic stomach syndrome was significantly higher in the whole-stomach group than in the gastric-tube group (95% CI: 0.17 to 0.55, *p* < 0.0001; Fig 7).

### Publication bias

**Fig 8:**
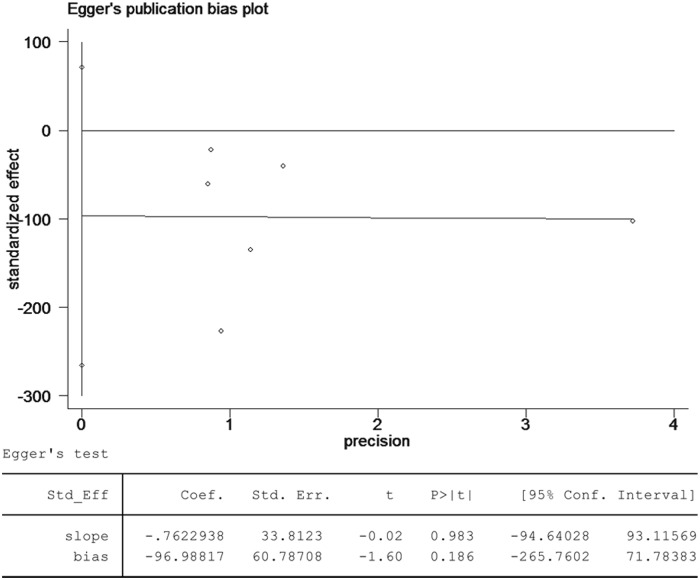
The Egger test for anastomotic leakage suggests that no publication bias is present in the pooled analysis.

The Egger test based on the data for anastomotic leakage suggested that there was no significant publication bias (*p* = 0.186; Fig 8).

## Discussion

Currently, esophagectomy is the primary treatment for the early and middle stages of esophageal cancer [3, 4]. Minimally invasive esophagectomy (MIE) could achieve similar long-term survival rates and reduce perioperative complications as compared with open esophagectomy (OE) [18, 19]. However, the change in the normal anatomical structure caused by esophagectomy (both MIE and OE) can lead to persistent gastrointestinal side effects, such as diarrhea, anorexia, nausea, acid regurgitation, and dysphagia [20]. In our clinical practice, we have found that many patients experience several episodes of intolerable gastrointestinal side effects after esophagectomy. Esophagogastric anastomosis via the gastric-tube approach more closely approximates the physiological form of the esophagus, and has been considered to reduce complications and improve the postoperative quality of life in many studies [7, 17,21]. Nevertheless, there is still some debate about the optimal reconstruction method after esophagectomy. The present meta-analysis therefore assessed six studies to provide the most comprehensive evidence about this argument.

Our meta-analysis showed that the rate of reflux esophagitis was significantly lower in the gastric-tube group than in the whole-stomach group. The following reasons might account for this finding: (1) The shape of the stomach tube (wide above and narrow below) is closer to the natural form of the esophagus, and can shorten the retention time of food in the stomach, which can reduce the incidence of reflux. Barbera et al. measured the gastric emptying rate by using a ^99m^Tc-labeled mixed solid–liquid meal, and found that food passed faster in the finer gastric-tube than in the whole-stomach [22]. (2) The oxyntic glands in the stomach, which are composed of parietal cells, chief cells, and mucous neck cells, are mainly distributed in the gastric corpus and gastric fundus. The reduction in gastric acid secretion after gastric-tube reconstruction can effectively prevent the occurrence of reflux esophagitis [23, 24]. (3) The volume of the tubular stomach is smaller than that of the whole stomach (21.4%–47.2% reduction) [25]. Thus, the compression of the stomach by the lungs during coughing or breathing is reduced, which can decrease the duration and amount of reflux [26].

The incidence of thoracic stomach syndrome was also significantly lower in the gastric-tube group than in the whole-stomach group. The main reason for this is the bigger stomach in the whole-stomach group, especially after eating [2]. A very large stomach compresses the lungs and mediastinum, which could restrict the recruitment of the lung on the surgical side [12]. Atelectasis might decrease lung ventilation and increase the risk of pulmonary infection.

No statistical differences were found in the rates of anastomotic leakage and anastomotic stenosis between the two groups. These findings may be explained the by following reasons: (1) Although the tubular stomach increased the arterial blood supply to the anastomosis, it did not improve the venous return and microvascular circulation, which might be more important for the occurrence of anastomotic leakage [27]. (2) The occurrence of anastomotic complications does not depend only on blood supply and anastomotic tension [28]; other factors (such as surgical approach, anastomotic method, gastric-tube size, and scar tissue hyperplasia) might also play a role. Zhang et al. compared the rates of anastomotic leakage between patients who had undergone esophagogastric anastomosis with hand suturing vs. stapling, and found that the incidence of anastomotic leakage was lower after stapling than after hand suturing [29]. Lerut et al. reported that the incidence of anastomotic stenosis was slightly higher after hand suturing than after stapling, especially in the case of double-layer anastomoses [30].

Our study has certain limitations. First, only 6 articles with 1571 patients were included in this study, and this might have affected the quality of the results. Second, the surgical technique (thoracolaparoscopic vs. open, two-field vs. three-field, hand suturing vs. stapling, etc.) was not uniform between the included articles. These differences might have affected the comparability of the data.

## Conclusion

Our analysis suggests that gastric-tube reconstruction can decrease the incidence of reflux esophagitis and thoracic stomach syndrome after esophagectomy for esophageal cancer. The rates of anastomotic leakage and anastomotic stenosis did not significantly differ between the two reconstruction methods. However, because of the significant heterogeneity across the studies and the inherent limitations of our meta-analysis, this conclusion should be validated through more large-scale, high-quality RCTs.

## Supporting information

S1 File PRISMA checklist. (DOC)

## References

[1] Jemal A, Bray F, Center MM, Ferlay J, Ward E, Forman D. Global cancer statistics. CA Cancer J Clin 2011; 61(2): 69–90. 10.3322/caac.20107 21296855

[2] Rice TW, Apperson-Hansen C, DiPaola LM, Semple ME, Lerut TE, Orringer MB, et al Worldwide Esophageal Cancer Collaboration: clinical staging data. Dis Esophagus 2016; 29(7): 707–714. 10.1111/dote.12493 27731549 PMC5591441

[3] Pauthner M, Haist T, Mann M, Lorenz D. Surgical Therapy of Early Carcinoma of the Esophagus. Viszeralmedizin 2015; 31(5): 326–330. 10.1159/000441049 26989387 PMC4789960

[4] Best LM, Mughal M, Gurusamy KS. Non-surgical versus surgical treatment for oesophageal cancer. Cochrane Database Syst Rev 2016; 3: CD011498 10.1002/14651858.CD011498.pub2 27021481 PMC7078786

[5] Rino Y, Yukawa N, Sato T, Yamamoto N, Tamagawa H, Hasegawa S, et al Visualization of blood supply route to the reconstructed stomach by indocyanine green fluorescence imaging during esophagectomy. BMC Med Imaging 2014; 14: 18 10.1186/1471-2342-14-18 24885891 PMC4041049

[6] Matsuda T, Kaneda K, Takamatsu M, Takahashi M, Aishin K, Awazu M, et al Reliable preparation of the gastric tube for cervical esophagogastrostomy after esophagectomy for esophageal cancer. Am J Surg 2010; 199(5): 61–64. 10.1016/j.amjsurg.2009.08.046 20202621

[7] Zhang M, Li Q, Tie HT, Jiang YJ, Wu QC. Methods of reconstruction after esophagectomy on long-term health-related quality of life: a prospective, randomized study of 5-year follow-up. Med Oncol 2015; 32(4): 122 10.1007/s12032-015-0568-0 25788030

[8] Li LJ, Wu QC, Zhang C, Zhang M, Qiang L, Jiang YJ, et al Comparison of long-term health-related quality of life in patients with different methods of reconstruction after oncologic esophagectomy. Zhonghua Yi Xue Za Zhi 2013; 93(35): 2790–2793. 24360173

[9] Collard JM, Tinton N, Malaise J, Romagnoli R, Otte JB, Kestens PJ. Esophageal replacement: gastric tube or whole stomach? Ann Thorac Surg 1995; 60(2): 261–267. 7646084 10.1016/0003-4975(95)00411-d

[10] Pierie JP, de Graaf PW, van Vroonhoven TJ. The vascularization of a gastric tube as a substitute for the esophagus is affected by its diameter. Dis Esophagus 1998; 11(4): 231–235. 10071804 10.1093/dote/11.4.231

[11] Boyle NH, Pearce A, Hunter D, Owen WJ, Mason RC. Intraoperative scanning laser Doppler flowmetry in the assessment of gastric tube perfusion during esophageal resection. J Am Coll Surg 1999; 188(5): 498–502. 10235577 10.1016/s1072-7515(99)00016-2

[12] Shu YS, Sun C, Shi WP, Shi HC, Lu SC, Wang K. Tubular stomach or whole stomach for esophagectomy through cervico-thoraco-abdominal approach: a comparative clinical study on anastomotic leakage. Ir J Med Sci 2013; 182(3): 477–480. 10.1007/s11845-013-0917-y 23397501

[13] Wells GA, Shea B, O'Connell D, Peterson D, J, Welch V, Losos M, et al The Newcastle-Ottawa Scale (NOS) for assessing the quality of non-randomised studies in meta-analyses. http://www.ohri.ca/programs/clinical_epidemiology/oxford.htm. Accessed 2012.

[14] Jadad AR, Moore RA, Carroll D, Jenkinson C, Reynolds DJ, Gavaghan DJ, et al Assessing the quality of reports of randomized clinical trials: is blinding necessary? Control Clin Trials 1996; 17(1): 1–12. 8721797 10.1016/0197-2456(95)00134-4

[15] Tabira Y, Sakaguchi T, Kuhara H, Teshima K, Tanaka M, Kawasuji M. The width of a gastric tube has no impact on outcome after esophagectomy. Am J Surg 2004; 187(3): 417–421. 10.1016/j.amjsurg.2003.12.008 15006575

[16] Li LJ, Wu QC, Zhang C, Zhang M, Qiang L, Jiang YJ, et al Randomized controlled study of esophagectomy with gastric tube reconstruction versus whole stomach reconstruction for esophageal cancer patients. Chinese Journal of Clinical Oncology 2009; 36(19): 1125–1127, 1131.

[17] Zhang R, Wang P, Zhang X, Zhang L, Li C. Gastric tube reconstruction prevents postoperative recurrence and metastasis of esophageal cancer. Oncol Lett 2011; 11(4): 2507–2509. 10.3892/ol.2016.4240 PMC4812516 27073507

[18] Guo W, Ma X, Yang S, Zhu X, Qin W, Xiang J, et al Combined thoracoscopic-laparoscopic esophagectomy versus open esophagectomy: a meta-analysis of outcomes. Surg Endosc 2016; 30(9): 3873–3881. 10.1007/s00464-015-4692-x 26659248

[19] Zhou C, Ma G, Li X, Li J, Yan Y, Liu P, et al Is minimally invasive esophagectomy effective for preventing anastomotic leakages after esophagectomy for cancer? A systematic review and meta-analysis. World J Surg Oncol 2015; 13: 269 10.1186/s12957-015-0661-z 26338060 PMC4560054

[20] Djärv T, Lagergren J, Blazeby JM, Lagergren P. Long-term health-related quality of life following surgery for oesophageal cancer. Br J Surg 2008; 95(9): 1121–1126. 10.1002/bjs.6293 18581441

[21] Zhang C, Wu QC, Hou PY, Zhang M, Li Q, Jiang YJ, et al Impact of the method of reconstruction after oncologic oesophagectomy on quality of life—a prospective, randomised study. Eur J Cardio-Thorac, 2011; 39(1): 109–114. 10.1016/j.ejcts.2010.04.032 20538475

[22] Barbera L, Kemen M, Wegener M, Jergas M, Zumtobel V. Effect of site and width of stomach tube after esophageal resection on gastric emptying. Zentralbl Chir, 1994; 119(4): 240–244. 8203175

[23] Bonavina L, Anselmino M, Ruol A, Bardini R, Borsato N, Peracchia A. Functional evaluation of the intrathoracic stomach as an oesophageal substitute. Br J Surg 1992; 79(6): 529–532. 1611444 10.1002/bjs.1800790618

[24] Domergue J, Veyrac M, Huin-Yan S, Rouanet P, Collet H, Michel H, et al pH monitoring for 24 hours of gastroesophageal reflux and gastric function after intrathoracic gastroplasty after esophagectomy. Surg Gynecol Obstet 1990; 171(2): 107–110. 2382185

[25] D'Journo XB, Martin J, Ferraro P, Duranceau A. The esophageal remnant after gastric interposition. Dis Esophagus 2008; 21(5): 377–388. 10.1111/j.1442-2050.2008.00849.x 18564166

[26] Chen SW, Fu XN, Xu CC, Zhang N, Pan XJ, Liu MY. Influence of Gastric Tube Reconstruction on Gastroesophageal Reflux after Esophagectomy for Esophageal Carcinoma. Acta Med Univ Med Tongji 2011; 40(5): 593–596.

[27] Panebianco V, Francioni F, Anzidei M, Anile M, Rolla M, Passariello R. Magnetic resonance- fluoroscopy as long-term follow-up examination in patients with narrow gastric tube reconstruction after radical esophagectomy. Eur J Cardiothorac Surg 2006; 30(4): 663–668. 10.1016/j.ejcts.2006.07.007 16945547

[28] Korenaga D, Toh Y, Maekawa S, Ikeda T, Sugimachi K. Intra-operative measurement of the tissue blood flow for evaluating blood supply to the gastric tube for esophageal reconstruction. Hepatogastroenterology 1998; 45(24): 2179–2180. 9951889

[29] Zhang XF, Shi XN, Han B. Hand-Suture versus Stapling Anastomosis in the Incidence of Anastomotic Leakage Following Esophagogastrostomy: A Systematic Review. Chin J Evid-Based Med 2012; 12(11): 1367–1371.

[30] Lerut TE, van Lanschot JJ. Chronic symptoms after subtotal or partial oesophagectomy: diagnosis and treatment. Best Pract Res Clin Gastroenterol 2004; 18(5): 901–915. 10.1016/j.bpg.2004.06.029 15494285

